# The Roles of PD-L1, Ki-67, P53, and Cyclin D1 in PitNETs: Diagnostic and Prognostic Implications in a Series of 74 Patients

**DOI:** 10.3390/ijms26167830

**Published:** 2025-08-13

**Authors:** Anna Krzentowska, Beata Biesaga, Ryszard Czepko, Anna Merklinger-Gruchała, Dariusz Adamek, Małgorzata Jasińska, Barbara Pluta, Wiktoria Michalska, Katarzyna Wróblewska, Filip Janczy, Filip Gołkowski

**Affiliations:** 1 Department of Endocrinology and Internal Medicine, Medical College, Andrzej Frycz Modrzewski Krakow University, 30-705 Kraków, Poland; fgolkowski@uafm.edu.pl; 2 Faculty of Health Sciences, Medical College, Andrzej Frycz Modrzewski Krakow University, 30-705 Kraków, Poland; bbiesaga@uafm.edu.pl (B.B.); amerklinger-gruchala@uafm.edu.pl (A.M.-G.); 3 Department of Neurosurgery, Medical College, Andrzej Frycz Modrzewski Krakow University, 30-705 Kraków, Poland; rczepko@uafm.edu.pl; 4 Department of Neuropathology, and Chair and Department of Pathomorphology, Faculty of Medicine, Jagiellonian University Medical College, 31-008 Kraków, Poland; mnadamek@cyf-kr.edu.pl; 5 Department of Histology, Jagiellonian University Medical College, 31-008 Kraków, Poland; malgorzata.m.jasinska@uj.edu.pl; 6 Students’ Scientific Interest Group, Medical College, Andrzej Frycz Modrzewski Krakow University, 30-705 Kraków, Poland; 66453@student-afm.edu.pl (B.P.); 66531@student-afm.edu.pl (W.M.); 69379@student-afm.edu.pl (K.W.); 72069@student-afm.edu.pl (F.J.)

**Keywords:** biomarkers, PD-L1, Ki-67, Cyclin D1, P53, PitNETs, transcription factors, invasiveness

## Abstract

Pituitary neuroendocrine tumors (PitNETs), also known as pituitary adenomas, are rare tumors that are usually benign. At present, the WHO PitNET classification based on transcription factors is in force. A problem is caused by invasive tumors and silent tumors which, despite a lack of obvious clinical symptoms, tend to behave aggressively. Factors influencing the clinical course of these tumors are currently being sought. The aim of our study was to assess the expression of programmed death-ligand 1 (PD-L1) and proliferation biomarkers (Ki-67, cyclin D1, and P53) in PitNETs depending on the transcription factor and adenoma subtype. The analysis was performed in seventy-four patients operated on in a single neurosurgical center for pituitary tumors. Immunohistochemistry was performed for transcription factors and biomarkers—PD-L1, Ki-67, P53, and cyclin D1—in tissue microarray format. Membranous expression of PD-L1 was scored as 0 (no expression) and ≥1%. Nuclear expression of Ki-67 was scored at <3% and ≥3%, and the expression of P53 and cyclin D1 was scored at <10% and ≥10%. The following tumors expressed PD-L1 at ≥1%: gonadotroph, 21 (28.4%); corticotroph, 5 (6.7%); gonadotroph/lactotroph, 2 (2.7%); null cell adenoma, 3 (4.0%); multiple synchronous PitNET, 2 (2.7%); immature PIT-1 tumor, 1 (1.3%); mature PIT-1 tumor, 1 (1.5%). Ki-67 ≥ 3% was found in the following PitNETs: gonadotroph, 3 (4.0%); corticotroph, 2 (2.7%); lactotroph, 1 (1.3%); multiple synchronous PitNET, 1 (1.3%); immature PIT-1 tumor, 1 (1.3%); and mature PIT-1 tumor, 1 (1.3%). Patients with Ki-67 ≥ 3% were statistically significantly younger (*p* = 0.03). All tumors (100%) with a combination of cyclin D1 ≥ 10% and P53 < 10% were invasive on the Hardy scale. Of the four factors, PD-L1 increased the odds of invasiveness the most (adjusted OR = 2.35; 95% CI: 0.56–9.90). PD-L1 expression was present in some types of PitNETs. PD-L1 expression may help in identifying null cell adenomas. High cyclin D1 with low P53 may indicate greater tumor invasiveness.

## 1. Introduction

The biological behavior of PitNETs is still unclear and is a challenge for clinicians. PitNETs account for approximately 10–15% of intracranial tumors [1,2,3]. At present, the WHO classification of PITNETs, which was proposed in 2017 and then modified in 2022, is in force [4,5]. The classifications are based on the immunohistochemical profile and transcription factors (TFs) from which the tumor originates (PIT-1, pituitary-specific POU-class homeodomain transcription factor; SF1, steroidogenic factor 1, and TPIT, T-box family member TBX19), which makes it possible to identify so-called silent PitNETs that do not produce clinically detectable amounts of hormones but have a specific molecular phenotype. This molecular stratification reflects the embryological origin and functional phenotype of a tumor and is clinically relevant due to its association with differences in tumor proliferation, hormone secretion, and aggressiveness [5]. The diagnosis and classification of PitNETs are based on clinical presentation, plasma hormone levels, magnetic resonance imaging (MRI) features (tumor size and cavernous sinus invasion), and histopathological features (immune subtype, Ki-67 index, mitotic count, and P53 positivity). Recently, a new prognostic clinicopathological classification (HYPOPRONOS) was proposed to predict the risk of recurrence of pituitary tumors [6]. The European Society of Endocrinology guidelines define an aggressive pituitary adenoma as a radiologically invasive tumor with an unusually rapid growth rate or as a tumor presenting with clinically relevant growth despite the optimal use of standard medical, surgical, and radiotherapeutic therapies [7]. According to various studies, about 2% of macroadenomas are aggressive in nature [8].

To date, many studies have been devoted to the subject of prognostic factors, thanks to which it is expected to be possible to determine the biological behavior of PitNETs. Studies have indicated that transcription-factor-defined subgroups may differ in the expression of key biomarkers, including programmed cell death protein 1 (PD-1; encoded by the *PSCD1* gene) and its ligand (PD-L1; encoded by the *CD274* gene), cyclin D1, tumor suppressor protein encoded by the *TP53* gene (P53), and the nuclear protein Ki-67 (Ki-67) [9,10,11].

These markers are involved in tumor proliferation (Ki-67), cell cycle control (cyclin D1), genomic instability (P53), and immune evasion (PD-L1), and their expression may be partially driven by the underlying differentiation program of the tumor. Therefore, analyzing these biomarkers in the context of transcription-factor-defined PitNET subgroups allows for a more nuanced understanding of tumor biology and may support better risk stratification and identification of candidates for targeted or immunomodulatory therapies. PIT-1-lineage tumors (e.g., somatotroph, lactotroph, thyrotroph) often exhibit distinct proliferative indices and may show variable P53 expression, potentially due to inherent differences in cell turnover and hormonal regulation [12]. SF1 tumors (gonadotroph lineage) are generally considered less aggressive but can occasionally exhibit high proliferation and P53 abnormalities, particularly in null cell variants [5]. TPIT tumors (corticotroph lineage), especially silent corticotroph tumors, are more frequently associated with aggressive behavior and may show elevated Ki-67 [13] and altered P53 status [14]. Null cell and multi-lineage tumors lack clear hormonal differentiation and are often associated with higher rates of atypical features and treatment resistance [4]. Investigating the expression of PD-L1, Ki-67, P53, and cyclin D1 within these transcription-factor-defined subgroups may provide insights into distinct biological behaviors of these tumors.

The engagement of the PD-1 receptor expressed on activated T cells and its ligand is a major co-inhibitory checkpoint signal that controls T-cell activities. Various types of cancers express high levels of PD-L1 and exploit PD-L1/PD-1 signaling to evade T-cell immunity [15]. The associations between PD-L1 expression and PitNET behaviors have been investigated in many studies [16,17,18,19,20]. Higher expression of PD-L1 has been demonstrated in somatotroph, lactotroph, and PIT-1-positive plurihormonal tumors [18], and lower expression in gonadotroph and corticotroph tumors [19]. A positive correlation of PD-L1 expression with tumor volume and cavernous sinus invasion has also been shown [20], but there are studies that did not confirm the correlation between PD-L1 and radiological features of invasion [19]. The roles of the tumor immune microenvironment (TIME) and the roles of macrophages and T cells are emphasized. Macrophages and T cells are principal immune infiltrates within the TIME. Multiple studies have confirmed that PD-1/PD-L1 and immune checkpoint inhibitors (ICIs) targeting PD-1/PD-L1 are of great importance in the treatment of numerous tumors [21]. Immune checkpoints are currently used in the treatment of pituitary tumors [22]. At present, immunotherapy is a subject of intensive research, as it is a promising method of treatment for aggressive PitNETs and rare pituitary carcinomas [23,24].

Ki-67 (encoded by the *MKI67* gene), which is a marker of cell proliferation, plays an important role in the assessment of pituitary tumors. This issue has been addressed in numerous studies [25,26,27,28]. It is present in the active phases of the cell cycle (G1, S, G2, M) and is absent in the resting phase (G0). It is expressed as the Ki-67 index; i.e., the percentage of cells showing positive expression. High Ki-67 (>3%) correlates with more frequent invasion of the cavernous sinus [28,29,30], recurrence after treatment [30,31,32], lower efficacy of pharmacological therapy, and the need for more intensive treatment, e.g., radiotherapy. The Ki-67 index helps to assess the risk of recurrence, tumor growth rate, and potential malignancy (when exceeding 10–20%). The cut-off for Ki-67 is assumed to be 3% [33]. In PIT-1-lineage tumors (lactotroph, somatotroph, thyrotroph), the typical Ki-67 value is <1–3%, which translates into low to moderate aggressiveness and risk of recurrence. In corticotroph tumors of the TPIT lineage, the Ki-67 value is 3–10% (sometimes over 10%), which results in high aggressiveness and a high risk of recurrence [27,34]. Silent corticotroph tumors are characterized by a Ki-67 index of >5–10% and, therefore, have an aggressive course and a high risk of recurrence. Crook cell tumors have similar indices [35,36]. Gonadotroph tumors of the SF1 lineage have a low KI-67 (i.e., <1–2%) and, therefore, a low risk of aggressiveness and recurrence. Null cell adenoma tumors usually have a Ki-67 value of 1–3%. Plurihormonal tumors show Ki-67 in the range of 3–10%, which may also be associated with increased aggressiveness. Pituitary carcinomas, in turn, show very high Ki-67 (i.e., >20–50%) and, therefore, very high aggressiveness and risk of recurrence and metastasis [32,37].

Cyclins play an important role in the regulation of cell progression through the cell cycle. Cyclin D1 is a regulatory protein (encoded by the *CCND1* gene) that plays a key role in the cell cycle, mainly in the transition from the G1 phase to the S phase and the initiation of DNA replication. The role of this protein in pituitary tumors has been extensively studied [38,39,40,41]. It activates cyclin-dependent kinases (CDKs). In pituitary tumors, cyclin D1 (CCND1) is particularly important, as it plays a role in the proliferation, progression, and potential aggressiveness of these tumors. Overexpression of cyclin D1 leads to excessive cell proliferation, resulting in rapid tumor growth. Cyclin D1 correlates positively with Ki-67. Higher cyclin levels may indicate a higher risk of residual tumor growth, recurrence after surgery [42,43,44], and invasion into the cavernous sinus [42,43]. Overexpression of this protein has been reported in a significant proportion of pituitary tumors (up to 54%), particularly in macroadenomas and hormonally inactive tumors [38,43,44].

The P53 protein is a nuclear transcription protein that acts as a tumor suppressor, monitoring DNA integrity and activating repair mechanisms or apoptosis in the event of damage [10,45]. In the 2004 WHO classification, the P53 protein was one of the three criteria for atypical pituitary adenoma. Although it is not currently a mandatory marker in routine PitNET diagnosis, it may be helpful in identifying potentially aggressive tumors. In a normal pituitary gland, P53 expression is very low; in most pituitary adenomas, it is minimal, while excessive (>10%) expression may indicate a more aggressive phenotype, a tendency to recur, and a propensity for invasion. Significant overexpression of P53 (strong diffuse expression in >10% of cells) may suggest a TP53 mutation, which may be associated with more aggressive clinical behavior of the tumor [46]. TP53 mutation has been described more frequently in highly proliferative, potentially metastatic PitNET types [47]. In addition, tumors with high P53 expression have been reported to show more frequent progression of residual pituitary adenoma [33] and invasion into the cavernous sinus [30].

In view of the above literature, we attempted to assess these four prognostic factors in our patient study group. We adopted the following goals:

1. Primary objective: Assessment of the expression of PDL1, Ki-67, cyclin D1, and P53 in PitNETs depending on the transcription factor and adenoma subtype.
2. Secondary objective: Assessment of the correlations between PDL1, cyclin D1, P53, and Ki-67 and tumor invasiveness, hormonal function, and parameters such as age, sex, maximum tumor size, and tumor volume.

## 2. Results

### 2.1. Patient Characteristics

The study cohort comprised 74 patients diagnosed with PitNETs. Briefly, the mean age at diagnosis was 57.4 years, with a standard deviation (SD) of 14.0 years and a median of 60.5 years (interquartile range [IQR]: 47.0–69.0). Females constituted 39.2% (n = 29) of the group, while males accounted for 60.8% (n = 45). With respect to tumor size, macroadenomas were predominant (97.3%), whereas giant adenomas (diameter > 40 mm) were present in 14.9% (n = 11). The mean tumor volume was 8.6 cm^3^ (SD 8.9), with a median of 5.0 cm^3^ (IQR: 3.1–10.2). Tumor invasiveness was evaluated using both the Knosp and Hardy classification systems. In the Knosp scale, grades 1 and 2 were classified as non-invasive, whereas grades 3 and 4 were considered invasive. Similarly, in the Hardy classification, tumors graded as stage 1 or 2 were deemed non-invasive, while a stage that was ≥3 indicated invasive growth. Based on these criteria, 51.4% (n = 38) of tumors were invasive according to the Knosp scale, and 77.0% (n = 57) were invasive according to the Hardy scale. Considering both radiological and histopathological findings, 78.4% (n = 58) of the tumors exhibited features of invasiveness. We divided the tumors into five groups according to the presence or absence of a transcription factor; i.e., tumors expressing Pit-1 only, SF1 only, or TPIT only, PitNETs with no distinct cell lineage = null cell adenoma, and tumors expressing more than two transcription factors (multiply PitNETs). PD-L1 expression was assessed in 66 patients (no data in 8 cases), Ki-67 expression was assessed in the overall group (n = 74), P53 expression in 67 (no data in 7 cases), and cyclin D1 expression in 70 patients (no data in 4 cases). The immunohistochemical (IHC) expression of PD-L1, cyclin D1, P53, and Ki-67 was evaluated in all available tumor samples. For PD-L1, the results were categorized as follows: 0%: negative cases, with no detectable PD-L1 expression in tumor cells; ≥1%: positive cases, with PD-L1 expression observed in ≥1% of tumor cells. The designation “missing” refers to cases where IHC staining could not be assessed due to the absence of tumor tissue in the analyzed section, typically resulting from tissue exhaustion or detachment during slide preparation. This classification applies to PD-L1, cyclin D1, and P53. These cases were excluded from the quantitative PD-L1 analysis, but are reported for transparency. In contrast, Ki-67 staining was successfully performed and interpretable in all cases, as all examined slides contained adequate tumor material. The detailed characteristics of the group are shown in Table 1.

### 2.2. Immunoexpression of PDL-1, Ki-67, P53, and Cyclin D1 and Their Correlations with Epidemiological, Clinical, and Histopathological Features

To analyze the relationships between all assessed epidemiological, clinical, and histopathological features and PDL-1, Ki-67, P53, or cyclin D1 expression, all tumors were divided into two subgroups—those with a lack of protein expression and those with protein overexpression—using specific cut-off points selected on the basis of data from the literature. In the case of PD-L1, Ki-67, P53, and cyclin D1, these cut-off points were at the levels of 1% for PD-L1 [18], 3% for Ki-67 [6,48], 10% for P53 [49], and 10% for cyclin D1. In the studied tumor cohort, we observed the presence of tumors with overexpression of PD-L1, Ki-67, P53, and cyclin D1 in 47.3%, 12.2%, 44.6%, and 39.2% of cases, respectively (Table 1).

To analyze the relations between all assessed epidemiological, clinical, and histopathological features, all tumors were divided into two groups—those with a lack of selective protein expression and those with overexpression—according to specific cut-off points. Next, we analyzed the differences between tumors with different expression of PD-L1. Among the analyzed subtypes, only tumors expressing PIT1 exclusively showed a statistically significant correlation with PD-L1 expression (*p* ≤ 0.05). Among tumors from the PIT1 lineage, positive PD-L1 expression (TPS ≥ 1%) was found in only one case, while in the remaining six, there was no PD-L1 expression, indicating an inverse relationship between the presence of PIT1 and PD-L1 expression. No significant differences were found for tumors from other cell lineages.

Nevertheless, when using a simplified classification into no expression (TPS 0%) and positive expression (TPS ≥ 1%), certain trends can be observed. Gonadotroph PitNETs, accounting for more than half of the entire group, showed positive PD-L1 expression (TPS >1%) in nearly 50% of cases. A similar trend was observed in corticotroph PitNETs, where PD-L1 was present in 62.5% of cases. In contrast, tumors such as somatotrophs, thyrotrophs, and most lactotroph tumors showed only a lack of PD-L1 expression.

Next, we analyzed the expression of proliferative proteins and their effects on various parameters of pituitary tumors. The results are presented in Table 2.

We analyzed the relationships between the expression of Ki-67, P53, and cyclin D1 and the expression of PD-L1. There was no statistically significant association between PD-L1 expression and the overexpression of Ki-67 (*p* = 0.71), P53 (*p* = 0.10), or cyclin D1 (*p* = 0.27); however, a trend toward higher P53 expression in PD-L1-positive cases was observed. The results are presented in Table 3.

In the case of PD-L1, the cut-off point was sought and differed for different types of PitNETs. In our study, we attempted to determine the cut-off point for this protein. We established the best cut-off point of TPS to maximize the sensitivity and specificity values for the different TFs and PitNET subtypes. The optimal cut-off point of TPS was ≥1.3% for tumors expressing SF-1. For tumors expressing TPIT, it was ≥5% while, for tumors from the PIT-1 lineage, the cut-off point was 0.00%. The results are presented in Table 4.

Analysis of cut-off points for transcription factors: TPS showed the highest effectiveness in predicting the presence of null cell adenoma, achieving an AUC of 0.75 and a sensitivity of 1.00 at a TPS threshold of ≥5%. In the ROC analysis, PD-L1 expression at TPS ≥ 5% best identified cases of null cell adenoma, achieving a sensitivity of 100% and specificity of 59%. In tumors expressing PIT-1, any detectable PD-L1 expression (TPS ≥ 1%) was associated with high sensitivity (0.86) and moderate specificity (0.58), with an AUC of 0.68. Thus, the presence of any PD-L1 expression (i.e., PDL1 ≥ 1%) was associated with a lower likelihood of the presence of a PIT-1 lineage. In the ROC analysis, the absence of PD-L1 expression (TPS = 0%) best identified PIT-1-positive cases, with a sensitivity of 85% and a specificity of 58%. This meant that the absence of PD-L1 expression was a relatively sensitive and moderately specific feature for the diagnosis of PIT-1-lineage tumors. For tumors of the SF1 lineage, the optimal TPS cut-off of ≥1.3% yielded only moderate sensitivity (56%) and low specificity (52%), which translated into a low AUC (0.57), indicating the limited usefulness of TPS in identifying this lineage. In the case of TPIT-positive tumors and those with multi-lineage differentiation (Multi-lineage), TPS also proved to be of little use, with AUC values of 0.50 and 0.59, respectively, meaning that PD-L1 levels did not allow these types of lesions to be effectively distinguished.

Analysis of cut-off points for individual PitNET types: In the ROC analysis, PD-L1 expression (TPS) showed varying diagnostic utility depending on the type of pituitary tumor. The best predictive properties were obtained for lactotroph PitNETs. The absence of PD-L1 expression (TPS = 0%) allowed for their diagnosis with perfect sensitivity (1.00) and moderate specificity (0.56), with an AUC of 0.78. This means that all lactotroph PitNETs did not express PD-L1, making TPS a very sensitive indicator of their presence. At the same time, the moderate specificity indicated that the absence of TPS also occurred in other types of tumors, albeit less frequently. Thus, the absence of PD-L1 expression suggested a lactotroph tumor, while its presence might make this diagnosis less likely.

In the case of immature PIT-1-lineage tumors, the absence of TPS also proved to be a relatively sensitive indicator, achieving a sensitivity of 0.80 and a specificity of 0.56 (AUC = 0.67). This means that most of these tumors did not express PD-L1, and its absence increased their likelihood of belonging to this category. However, the moderate specificity indicates the limited ability of TPS to distinguish these tumors from others.

For gonadotroph PitNETs, the optimal cut-off point for PD-L1 expression (TPS) was TPS ≥ 1.3%. At this threshold, the sensitivity was 55% and the specificity was 50%, with an AUC of 0.54. Thus, PD-L1 expression above 1.3% allows for the detection of slightly more than half of gonadotroph cases (moderate sensitivity) but, at the same time, it occurs equally often in other types (low specificity). An AUC value very close to 0.5 indicated that TPS in this range practically did not distinguish gonadotroph tumors from other types of tumors. Therefore, TPS has very limited usefulness as a diagnostic marker for this type of adenoma.

In corticotroph PitNETs, TPS ≥ 5% allowed for diagnosis with moderate sensitivity (0.63) and low specificity (0.59), with an AUC of only 0.50. This means that PD-L1 expression did not effectively differentiate these tumors from other types. PD-L1 appeared with similar frequency in both groups, making TPS an unsuitable diagnostic indicator in this case.

**Figure 1:**
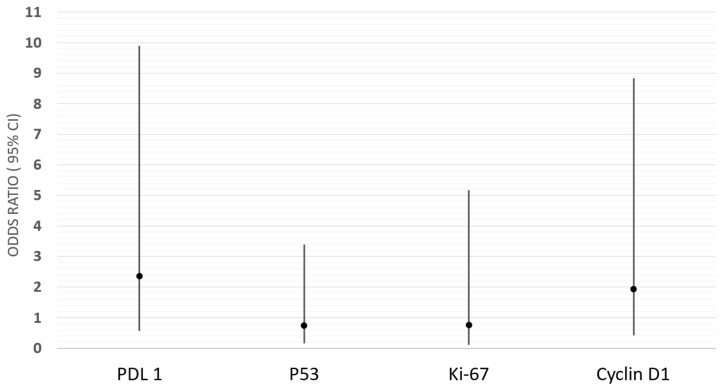
Results of the multivariate logistic regression analysis: odds ratios (ORs) and 95% confidence intervals (CIs) for biomarkers (PD-L1, P53, Ki-67, cyclin D1) in association with tumor invasiveness. Model fit statistics: Hosmer–Lemeshow χ^2^ = 3.63, *p* = 0.60; AUC = 0.64 (SE = 0.10); Cox–Snell R^2^ = 0.04; Nagelkerke R^2^ = 0.06.

Next, we conducted a univariate and multivariate analysis of the impacts of all four factors (PD-L1, P53, Ki67, and cyclin D1) on the invasiveness of pituitary tumors. The results are presented in Figure 1.

In the logistic regression analysis, none of the evaluated biomarkers (PD-L1, P53, Ki-67, cyclin D1) reached statistical significance in association with tumor invasiveness defined by Knosp grade B or Hardy grade D (all *p* > 0.05). However, the crude analysis revealed a promising trend for PDL1 expression, which was associated with odds of invasiveness that were increased by more than twofold (crude OR = 2.52; 95% CI: 0.67–9.43; *p* = 0.17). After adjusting for other biomarkers (P53, Ki-67, cyclin D1), the association remained elevated (adjusted OR = 2.35; 95% CI: 0.56–9.90), but this result still did not reach statistical significance (*p* = 0.25). Similarly, cyclin D1 seemed to increase the odds of invasiveness in both the crude (crude OR = 1.34; 95% CI 0.39–4.52) and adjusted model (adjusted OR = 1.93; 95% CI 0.42–8.84), but the results were not statistically significant (*p* = 0.40), which may have been due to the small sample size. The overexpression of other biomarkers (Ki-67 and P53) showed weaker and protective associations with tumor invasiveness, as well as non-significant associations both before and after the adjustment (*p* = 0.78, *p* = 0.70, respectively, for Ki-67 and P53). These results remained essentially unchanged after additional adjustments for age and sex. The Hosmer–Lemeshow test indicated a good model fit (χ^2^ = 3.63; *p* = 0.60). The model’s discriminative ability, assessed using the area under the ROC curve, was moderate (AUC = 0.64; SE = 0.10). The pseudo-R^2^ values also indicated limited explanatory power (Cox–Snell R^2^ = 0.04; Nagelkerke R^2^ = 0.06).

### 2.3. Combined Analysis of Ki-67 and P53 Expression or Ki-67 and Cyclin D1 Expression

#### 2.3.1. Combined Analysis of Ki-67 and P53 Factors

We divided the entire group into different combinations of pairs of Ki-67 and P53 factors, i.e., Ki ≥ 3 & P53 ≥ 10 (1), Ki ≥ 3 & P53 < 10 (2), Ki < 3 & P53 ≥ 10 (3), and Ki < 3 & P53 < 10 (4), and we found that the median ages in these groups were as follows: for group 1, 41 years (36–51); for group 2, 48.5 years (35–62); for group 3, 63 years (50–68); for group 4, 62 years (53–70.5). Group 1 appears to be younger than the others; however, the differences did not reach statistical significance after correction for multiple comparisons (overall Kruskal–Wallis test: *p* = 0.04; post hoc comparisons: group 1 vs. 3—*p* = 0.06; group 1 vs. 4—*p* = 0.06), indicating a trend that warrants further investigation. With respect to other parameters—i.e., sex, tumor size and volume, transcription factor type, hormonal function, and invasiveness—no statistically significant differences were found. The distribution of these combinations depending on the PitNET type is presented in Table 5.

#### 2.3.2. Combined Analysis of Cyclin D1 and P53 Factors

A division into pairs of factors was also performed for cyclin D1 and P53: Cyk ≥ 10 & P53 ≥ 10 (1), Cyc ≥ 10 & P53 < 10 (2), Cyc < 10 & P53 ≥ 10 (3), and Cyc < 10 & P53 < 10 (4). The relations to the parameters age, sex, tumor size and volume, transcription factor type, and hormonal function were not found to be statistically significant. The distribution of these combinations of factors depending on the type of PitNETs is presented in Table 6.

#### 2.3.3. Analysis of the Effect of the Combination of Ki-67, P53, and Cyclin D1 on the Invasiveness of PitNETs

In addition, the incidence of invasive tumors (according to the Hardy scale) was analyzed in four subgroups based on Ki-67 and P53 expression (Ki ≥ 3 & P53 ≥ 10, Ki ≥ 3 & P53 < 10, Ki < 3 & P53 ≥ 10, Ki < 3 & P53 < 10) or cyclin D1 and P53 expression levels (Cyc ≥ 10/P53 ≥ 10, Cyc ≥ 10/P53 < 10, Cyc < 10/P53 ≥ 10, Cyc < 10/P53 < 10). This analysis showed that all tumors (100%) in the group with high cyclin D1 expression and low P53 expression (n = 10) were invasive. For the remaining three combinations of protein expression (n = 52), the percentage of invasive tumors was 75%. Although a clear trend was observed, the difference did not reach statistical significance (exact Fisher’s test: *p* = 0.10). The results are presented in Table 7.

## 3. Discussion

Pituitary neuroendocrine tumors are still a major challenge for clinicians. Key problems include their different biological behaviors, tendency to be invasive, tendency to relapse and, in some cases, their potential for aggressive behavior. The transcription-factor-based classification of PitNETs provides a biologically meaningful framework that corresponds to tumors’ developmental lineage and hormonal profile. This molecular subtyping—distinguishing PIT-1 (somatotroph, lactotroph, thyrotroph), SF1 (gonadotroph), TPIT (corticotroph), and null cell and multi-lineage tumors—has been associated with differences in proliferation rates, recurrence risk, and treatment responsiveness [5]. Markers such as Ki-67 and P53 are established indicators of proliferative potential and tumor aggressiveness [27,34,35], while cyclin D1 is directly involved in cell cycle progression [44]. PD-L1 expression, in turn, may reflect a tumor’s ability to escape immune surveillance and could serve as a predictive biomarker for possible immunotherapy [21]. Analyzing these markers across TF- defined subtypes enables the identification of biologically distinct PitNET subsets, including those with potentially more aggressive behavior (e.g., silent TPIT-positive tumors) or immunologically active profiles. This approach is clinically relevant, because it offers the possibility of risk assessment and the possibility of target therapies.

It has also been emphasized in research that the tumor immune microenvironment (TIME) may play a role in the different behavior of tumors. It could determine the biological and clinical behavior of a neoplasm and may have prognostic implications. Macrophages and T cells are principal immune infiltrates within the TIME. In a study by Vela-Patiño et al., characteristic expression profiles of genes associated with the immune system, including those encoding interleukins and chemokines, were identified for each tumor lineage [50].

Different subtypes of PitNETs display distinct immune patterns, influencing tumor progression behaviors. PD-L1 on tumor cells binds to programmed cell death 1 (PD-1) on immune cells, contributing to tumor immune escape [51]. However, PD-L1 expression is regulated by various factors, leading to different meanings of PD-L1 positivity. The presence of PD-L1 in pituitary tumors may suggest potential sensitivity to immunotherapy (e.g., PD-1/PD-L1 inhibitors). When analyzing the results obtained in our study, it should be emphasized that the results may have been influenced by the small size of the patient group studied, as well as by the large quantitative disparities between individual types of PitNETs. Positive PD-L1 expression (≥1%) was found in 53% of patients. In our group of patients, only in the case of PIT1 tumors was a statistically significant correlation with PD-L1 expression found (*p* ≤ 0.05); i.e., an inverse correlation between the presence of PIT1 and PD-L1 expression was demonstrated. When analyzing individual types of PitNETs using a simplified division into no expression (TPS 0%) and positive expression (TPS ≥ 1%), certain trends were observed: gonadotropic tumors, which accounted for more than half of the entire group, showed positive PD-L1 expression (TPS > 1%) in nearly 50% of cases. A similar trend was observed in corticotropic tumors, where PD-L1 was present in 62.5% of cases. In contrast, tumors such as somatotrophic, thyrotrophic, and most lactotrophic tumors did not show PD-L1 expression; however, it should be noted that these tumor types were isolated cases in our study. This fact may explain the inconsistency with the results of other studies [18].

In our study, PD-L1 expression was more pronounced in null cell adenomas, while its diagnostic value in other PitNET subtypes appeared limited. It should be noted that in our study group, TPS showed the highest effectiveness in predicting the presence of null cell adenoma. This observation may be explained by several biological and technical factors. Null cell adenomas, lacking hormonal and lineage-specific marker expression, represent a less differentiated tumor subtype. This may be associated with an altered TIME and higher expression of immune checkpoint molecules, such as PD-L1, as part of immune evasion mechanisms [52]. In the study by Wang et al. it was shown that pituitary tumors show the so-called 3 immune clusters [52]. It has been shown that inactive pituitary tumors (NF-PitNETs), including null cell adenomas, can have a more immunoactive environment than hormonally active tumors, and thus PitNETs are characterized by differential immunoinfiltration [53].

The predictive specificity of PD-L1 for null cell adenomas likely arises from a unique interplay between their undifferentiated cellular identity and tumor–immune interactions. The lack of differentiation may be associated with a more “immune-cold” or “immune-evasive” phenotype, prompting upregulation of immune checkpoint molecules such as PD-L1 as a compensatory mechanism. This contrasts with more differentiated PitNET types, which may engage different immune modulation pathways [20]. On the other hand, the tumor microenvironment in hormone-producing adenomas often exhibits lower immune cell infiltration—particularly of cytotoxic T lymphocytes—compared with more aggressive or undifferentiated tumors [54,55]. Immune cells within the PitNET microenvironment have the potential to release various cytokines and chemokines, and in turn can modify the biological behavior of these tumors [56]. Null cell adenoma has been shown to be more invasive and have a more aggressive clinical course compared to gonadotroph tumors, i.e., clinically silent tumors [57]. Null cel adenoma were more likely to show a tendency to invade the sphenoid sinus [57,58] and a tendency to recur [58]. Additionally, null cell tumors have been reported to display a higher proliferative index and more aggressive clinical behavior compared with hormone-producing PitNETs [59]. These studies indicate that these tumors, although clinically silent, are a diagnostic and therapeutic problem. In contrast, the generally low and heterogeneous PD-L1 expression observed in more differentiated PitNETs limits its diagnostic or prognostic applicability in those subtypes. It should be noted that methodological factors such as focal staining, tissue sampling variability, or technical differences in staining protocols may also reduce detection sensitivity in low-expressing tumors. These findings suggest that PD-L1 expression may have selective diagnostic relevance, particularly in less differentiated tumors such as null cell adenomas.

When analyzing PD-L1 expression according to hormonal activity, no statistically significant differences were found in our study, as in other studies [60]. However, there are studies confirming more frequent positive PD-L1 expression in hormonally active tumors [22,61,62]. These differences may result from the nature of the group analyzed. Referring to the impact of PD-L1 on invasiveness based on multivariate analysis, our study showed that PD-L1 was the strongest factor influencing invasiveness (OR = 2.35; 95% CI: 0.56–9.90) among the factors studied; however, the results were not statistically significant. A similar effect of PD-L1 on invasiveness has been described in the literature [20,22], but there are studies that did not confirm this relationship [19]. In our study, tumors with positive PD-L1 expression had a larger volume and maximum dimension, but this was not statistically significant. Similarly, other studies have not shown a statistically significant relationship between PD-L1 expression and tumor size [19]. In summary, based on our study, it can be said that the relationships between PD-L1 and size, volume, and invasiveness remain controversial.

Although our findings suggest that PD-L1 expression may be associated with certain tumor subtypes and invasive behavior, its role as a clinically actionable biomarker in PitNETs remains uncertain. The observed trends should be interpreted with caution, as the current evidence does not yet support the routine use of PD-L1 status in guiding immunotherapeutic strategies in these tumors. Therefore, further prospective studies, including functional and therapeutic validation, are needed to determine whether PD-L1 expression has predictive or prognostic value in this context.

When analyzing the results for Ki-67 in our study, it should be noted that patients with a Ki-67 index of ≥3% were significantly younger, which may suggest a more aggressive biological behavior in this group. Although a larger tumor size and volume were also observed in these patients, the differences were not statistically significant. These findings are partially consistent with the study of Wang et al., who indicated that younger age, rich tumor vascularity, and dorsum sellae erosion may be predictive of higher Ki-67 values [63]. Furthermore, a Ki-67 index of <3% was predominant in tumors expressing PIT-1, SF1, and TPIT, as well as in all null cell adenomas. A Ki-67 index of ≥3% appeared only in isolated cases across different PitNET subtypes, including corticotroph, lactotroph, and PIT-1-lineage tumors. It should also be noted that Ki ≥ 3% was also found in three cases of gonadotroph PitNETs. No significant association was found between Ki-67 expression and tumor invasiveness or hormonal activity, which is in line with previous findings [8]. Although Ki-67 is widely used as a marker of cellular proliferation and is included in the assessment of pituitary neuroendocrine tumors (PitNETs), its predictive value for tumor behavior remains controversial.

This finding is consistent with previous reports suggesting that the Ki-67 index, while useful in identifying atypical or potentially aggressive tumors, does not reliably correlate with clinical features such as invasiveness or hormone secretion in the broader spectrum of PitNETs [1,27,64,65]. One possible explanation is the considerable biological heterogeneity of PitNETs, including intratumoral variation in proliferative activity. Moreover, sampling limitations in immunohistochemical analysis may lead to underrepresentation of the most proliferative areas.

According to the 2017 WHO classification of endocrine tumors, Ki-67 can aid in risk stratification, particularly when used alongside other markers such as mitotic count and P53 expression, but should not be used in isolation to predict clinical behavior. This perspective is supported by meta-analyses and long-term follow-up studies, which demonstrate that Ki-67 has limited prognostic value when considered independently [12,66].

With regard to the P53 protein, no significant effect on various tumor parameters was observed in our group. The literature also does not confirm the effect of P53 on tumor size and recurrence risk, but a link between this protein and cavernous sinus invasion has been confirmed [30,64].

Cyclin D1 is an important cell cycle regulator and plays an important role as an oncoprotein in tumor proliferation. High levels of cyclin D1 are required to sustain tumor growth [43]. In our study, multivariate analysis showed that PD-L1 and cyclin D1 are factors that increase the odds of tumor invasiveness (OR (95% CI) 2.35 (0.56–9.90) and 1.93 (0.42–8.84), respectively). The lack of statistical significance may result from the small size of the study group. In addition, in our study, a cyclin D1 expression of ≥10% was more common in hormone-inactive tumors (45.45%) than in hormone-active tumors (26.67%), which was also confirmed in another study [43]. With regard to individual transcription factors and PitNET types, it was found that they were more often characterized by a cyclin expression of <10%.

The combined analysis of cyclin D1 and P53 expression showed that all tumors (100%) in the group with high cyclin D1 expression and low P53 expression (n = 10) were invasive, while the combined analysis of Ki-67 and P53 expression did not yield a distinct molecular or clinicopathological subgroup within the analyzed cohort (except age). This finding suggests a potential synergistic role of cyclin D1 upregulation and P53 downregulation in driving tumor invasiveness, which may not be captured through Ki-67/P53 co-expression patterns alone. While Ki-67 is widely used as a general proliferation marker due to its expression across all active phases of the cell cycle (G1, S, G2, and M) and absence in quiescent (G0) cells, it does not provide mechanistic insights into the regulation of cell cycle entry or progression. In contrast, cyclin D1 functions as a regulatory protein that plays a direct and active role in driving cell cycle progression through the G1 phase by activating cyclin-dependent kinases (CDK4/6), leading to phosphorylation of the retinoblastoma protein (pRb) and subsequent E2F-mediated transcription of S-phase genes. As such, cyclin D1 not only reflects proliferative activity but also indicates upstream signaling events involved in cell cycle control, including those mediated by mitogenic stimuli or oncogenic pathways. These results also underscore the limitations of relying solely on proliferation and TP53 status for tumor stratification and support the utility of incorporating cell cycle regulators such as cyclin D1 into biomarker panels for improved prognostic or predictive accuracy. Further validation in larger independent cohorts is warranted.

In our study, there were also tumors with an increased potential for aggressive behavior. There were the following tumors: two cases of Crook tumors, two cases of silent corticotroph tumors, three cases of plurihormonal PIT-1-positive tumors, and two cases of male lactotroph tumors. According to the literature, these tumors are characterized by Ki-67 > 3% (often >10%) and mitotic activity of >2/10HPF [8]. A number of studies have confirmed high values of Ki-67 in aggressive silent corticotroph tumors [67,68] and Crooke’s cell adenoma [69]. In our study group, however, these tumors showed a very diverse distribution of the biomarkers studied. In addition, in our study, three cases of gonadotroph tumors with Ki ≥ 3% were found, which confirms that tumors of this type may also tend to behave aggressively, which is also described in the literature [7,8].

This study has several limitations that should be acknowledged. The most significant is the relatively small size of the study group, particularly within the rarer subtypes of PitNETs, which may have limited the statistical power to detect significant associations. Additionally, the uneven distribution of tumor types reduced the possibility of subgroup analyses and generalization of findings. Therefore, the results should be interpreted with caution. Correlation analyses were conducted based on available data for each biomarker pair. As a result, not all analyses were based on the exact same set of cases, which may introduce variability in the interpretation of biomarker associations and witch should also be considered a limitation of our study. However, given the low proportion of missing data, we consider this limitation to have a minimal impact on the overall findings. Despite these limitations, the study has important strengths. It provides a comprehensive analysis of both histopathological and immunohistochemical features, including transcription factors, pituitary hormones, and biomarkers such as PD-L1, cyclin D1, Ki-67, and P53, integrated with radiological tumor characteristics. This multifaceted approach allows for a better understanding of the biological behaviors of PitNETs and contributes new data on the diagnostic and potential prognostic value of selected biomarkers.

## 4. Materials and Methods

### 4.1. Patients

Retrospective immunohistochemical and radiological analysis of pituitary tumors was performed. The study included a group of seventy-four patients who underwent surgery at St. Raphael’s Hospital in Krakow, Poland between 2022 and 2024, who were referred for surgery for a tumor within the sella turcica, and in whom a pituitary adenoma was subsequently confirmed via histopathology (HP). Each patient gave informed consent for the collection of tumor tissue for the study. The patient data were anonymized. The study was approved by the Bioethics Committee of Andrzej Frycz Modrzewski Krakow University (permission No. KBKA/31/O/2024 issued on 6 June 2024).

### 4.2. Classification of Pituitary Neuroendocrine Tumors (PitNETs)

The pituitary neuroendocrine tumors (PitNETs) included in this study were classified according to the 2022 World Health Organization (5th edition, Website beta version 2022) classification of endocrine and neuroendocrine tumors. The classification was based on immunohistochemical (IHC) staining for pituitary hormones (GH, PRL, ACTH, FSH, LH, TSH) and the expression of lineage-specific transcription factors (PIT1, SF1, and TPIT). Tumors were categorized into the following lineages and subtypes: PIT1 lineage: somatotroph, lactotroph, thyrotroph, and plurihormonal PIT1-positive tumors; TPIT lineage: corticotroph tumors; SF1 lineage: gonadotroph tumors; null cell tumors: those lacking both hormone and transcription factor expression. Only tumors with available and interpretable IHC results for both hormonal and transcription factor markers were included in the subtype analysis. The classification results are summarized in Table 1.

### 4.3. Immunohistochemical Assessment

#### 4.3.1. Immunohistochemical Assessment of the Hormones and Transcription Factors

The postoperative materials from the resected tumors were examined histopathologically. Immunohistochemical evaluation included the levels of pituitary hormones (ACTH, GH, PRL, TSH, LH, FSH) and transcription factors (Pit-1, SF1, and TPIT). Based on the hormones secreted by the adenoma and the clinical picture, the tumors were classified as either hormonally active or inactive. Sections of 3 μm were used. After routine deparaffinization, rehydration, and blocking of endogenous peroxidase activity freshly made 3% H_2_O_2_ in methanol for 20 min at room temperature, sections were microwaved for 30 min using EDTA buffer (pH = 9.0) for antigen retrieval and then incubated with primary antibody (ready-to-use primary antibody: Anti-ACTH AM487-5M mouse monoclonal (clone: AH26), Anti-Prolactin AM978-5M mouse monoclonal (clone: PRL/2644), Anti-FSH-Beta AM986-5M mouse monoclonal (clone: FSH b/1062), Anti-Thyroid-Stimulating Hormone mouse mono-clonal AM033-5M (clone: 5404), Anti-Luteinizing Hormone AN787-5M mouse monoclonal (clone: SP132)) for 30 min at room temperature. This was followed by Labeled Polymer-HRP anti-mouse (peroxidase-labeled polymer conjugated to goat anti-mouse immunoglobulins in Tris-Hcl buffer containing stabilizing protein) for 30 min at room temperature with DAB (di-aminobenzidine tetrahydrochloride) as chromogen was applied for 8 min at room temperature. Slides were counterstained with Mayer hematoxylin for 30 s. In the cases of GH, PIT1, SF1, and TPIT, citrate buffer with a pH of 6.0 was used for antigen retrieval, and then slides were incubated with an Ultra Vision Quanto Detection System (V-TL-125-QHD) containing blocking serum (Ultra Vision Protein Block) to minimize nonspecific background staining with 5 min of incubation. For GH and the transcription factors, the following antibodies were used: Anti-human Growth Hormone AR707-5R polyclonal rabbit (ready to use with incubation for 30 min at room temperature), Anti-TPIT ab243028 mouse monoclonal (clone: CL6251) at a 1:1000 dilution, Anti-Pit-1 ab272639 rabbit polyclonal at a 1:1000 dilution, and Anti-SF1 antibody ab217317 rabbit monoclonal EPR19744 at a 1:1500 dilution with incubation for 60 min at room temperature. This was followed by the application of the Primary Antibody Amplifier Quanto with 10 min of incubation; subsequently, the antibody was conjugated with HRP-labeled polymer (HRP Polymer Quanto) with 10 min of incubation, and DAB (diaminobenzidine tetrahydrochloride) was used as chromogen was applied for 8 min at room temperature. Slides were counterstained with Mayer hematoxylin for 30 s. To confirm the specificity of the primary antibody, positive and negative control tests were performed according to the manufacturer’s instructions. The antibodies of the following manufacturers were applied (used): (Anti) ACTH, LH, TSH, Prolactin: BioGenex; (Anti) GH: Bio SB Inc.; (Anti) FSH: BOND; (Anti) Pit-1 TPIT, SF-1: Abcam. Sections of human pituitary gland tissue were included as a positive control. The negative control test included substitution of the primary antibody with phosphate-buffered saline with a pH of 7.4.

#### 4.3.2. Immunohistochemistry of PD-L1, Cyclin D1, Ki-67, and P53

Formalin-fixed, paraffin-embedded (FFPE) tumor tissue blocks were sectioned with a thickness of 4 µm using a rotary microtome. Sections were mounted on Superfrost Plus™ glass slides (Gerhard Menzel, Glasbearbeitungswerk GmbH & Co. KG, Braunschweig, Germany) and dried overnight at 37 °C, followed by baking at 60 °C for 1 h prior to staining. Slides were deparaffinized in three changes of xylene (5 min each), followed by rehydration through a graded ethanol series (2 × 100% and 2 × 95%). Heat-induced epitope retrieval was performed using citrate buffer (Target Retrieval Solution, (pH = 6.1, DAKO Cytomation, Glostrup, Denmark)), at 96 °C for 50 min. Slides were allowed to cool gradually to room temperature (RT) in the buffer and then rinsed in distilled water. To quench endogenous peroxidase activity, sections were incubated in 3% hydrogen peroxide (H_2_O_2_) and in methanol for 30 min. For non-specific protein binding, slides were preincubated with eBioscience™ IHC/ICC Blocking Buffer—High Protein (Thermo Fisher Scientific) for 5 min at RT in a humidified chamber. Whole-night incubation with diluted primary antibodies was carried out at 4 °C in a humidity chamber. The following antibodies were used: PD-L1 polyclonal antibody (Invitrogen, dilution: 1:100, Thermo Fisher Scientific, Waltham, MA, USA), Ki-67 Antigen, Clone MIB-1 (Dako Denmark A/S, Glostrup|Denmark, dilution: 1:100), P53, clone DO-7 mouse antibody (Cell Signalling BioTechnology, Danvers, MA, USA, dilution: 1:100,), and anti-cyclin D1/BCL-1 (Thermo Fisher Scientific, Waltham, MA, USA, clone SP4, dilution 1:50). In all cases, the reaction was visualized using a BrightVision system (Immunologic, Duiven, The Netherlands) and 0.01% 3.3-diaminobenzidine tetrahydrochloride (Vector Laboratories, Inc., Burlingame, CA, USA). Slides were counterstained with Mayer’s hematoxylin for 1 min, rinsed in running tap water for 10 min, dehydrated through graded alcohols, cleared in xylene, and coverslipped using a permanent mounting medium (e.g., DPX). Each staining batch included a known PD-L1-positive control (e.g., tonsil or placenta tissue) and a negative control (primary antibody omitted).

**Figure 2:**
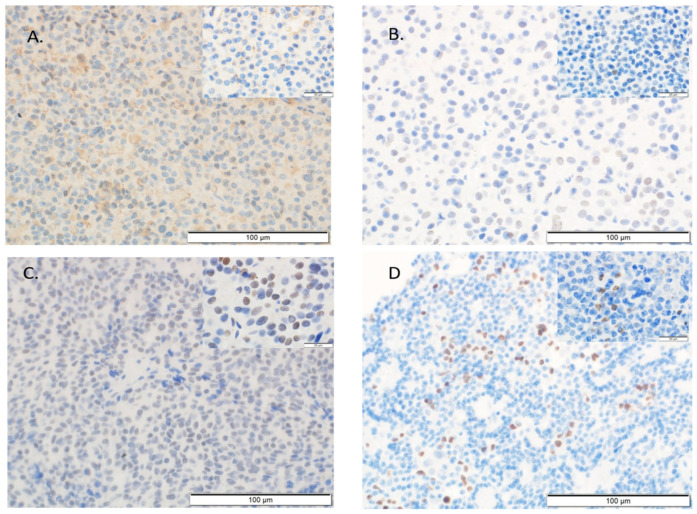
Microphotographs of immunohistochemical staining of pituitary adenocarcinoma. (**A**) Membranous staining for PD-L1. (**B**) Nuclear staining for Ki-67. (**C**) Nuclear staining for P53. (**D**) Nuclear staining for cyclin D1. Magnification: 400×. In the upper-right corner of each micrograph, 1000× magnification is shown to visualize individual positively stained cells.

All evaluations were performed blinded to the study endpoint. In case of PD-L1 expression, as in other studies [18], membranous staining of tumor cells was considered positive (Figure 2A), regardless of intensity, and it was scored using the Tumor Proportion Score (TPS), defined as the percentage of viable tumor cells showing partial or complete membrane staining at any intensity. In our study, only membranous PD-L1 staining—defined as continuous or partial staining along the plasma membrane of tumor cells—was considered positive and scored in accordance with current clinical IHC scoring guidelines. Cytoplasmic staining without clear membrane localization was not scored, as its biological relevance remains uncertain.

For Ki-67, the P53 and cyclin D1 expression labeling index was analyzed, which indicates the percentage of tumor cells with positive nuclear staining (Figure 2B–D).

The cut-off points for PD-L1, Ki-67, and P53 expression and the cyclin D1 expression/or the lack thereof were assumed to be at the levels of ≥1%, 3%, 10%, and 10%, respectively. For PD-L1, we used the Tumor Proportion Score (TPS) with a ≥1% cut-off, which is consistent with prior studies investigating PD-L1 expression in pituitary tumors [18]. The Ki-67 cut-off value of ≥3% is based on previous reports that identified this threshold as potentially relevant for distinguishing more proliferative or aggressive PitNETs [7,48]. For P53, we evaluated nuclear staining and used an empirical semi-quantitative scale, in line with other PitNET studies [49]. Cyclin D1 was scored using the method described by Liu et al. [70]. There is no uniform, universally accepted threshold for cyclin D1 in PitNET in the literature.

#### 4.3.3. Magnetic Resonance Imaging of the Tumors

Each patient was subjected to a pituitary-targeted magnetic resonance imaging (MRI) scan before surgery; in individual cases, a computer tomography (CT) scan of the head was performed due to the fact that MRI was contraindicated. Radiological assessment was based on pituitary MRI with contrast. The thickness of the layers used was 2–3 mm. All standard MRI sequences necessary to diagnose pituitary adenoma were used. Measurements were performed using contrast-enhanced T1-weighted images. A volumetric method was used based on standard software available in the Osirix browser. Based on the MRI image, the tumor was measured in 3 dimensions, i.e., AP, ML, and CC (cor × sag × cc), and the tumor volume was calculated. In addition, tumor invasion into the cavernous sinuses was assessed using the Knosp scale, while the invasion towards the sella turcica was assessed according to the Hardy scale. The Knosp classification system is used as follows Grade 0: The tumor does not cross the medial line of the internal carotid artery; grade 1: the tumor is confined medial to the intercavernous line, crossing the vertical meridian of the carotid siphon in the cross-section; grade 2: tumors extend past the intercavernous line but stay within the line tangent to the supracavernous and intracavernous carotid arteries; grade 3: tumors spread lateral to the lateral tangential line; grade 4: tumors totally encase the intracavernous carotid artery. In the Hardy scale, the relation of the pituitary tumor to the sella is defined using grades I to V; intrasellar microadenoma is grade I; macroadenoma causing diffuse enlargement but no perforation of the sellar floor is grade II; those causing focal eruption through the anterior sella surface are grade III; those causing extensive destruction into the sphenoid sinus are grade IV; those that exhibit CSF (cerebrospinal fluid) or hematogenous spread are grade V. A tumor with suprasellar or parasellar extension is further designated using stages 0 and A to E. Stage 0: tumors are intrasellar; stage A: tumors reach only the suprasellar cistern; stage B: tumors encroach upon the anterior recesses of the third ventricle; stage C: tumors elevate the floor of the third ventricle; stage D: tumors extend to intradural intracranial growth; stage E: tumors invade the cavernous sinus laterally.

In our study, tumors of Knosp grades 1 and 2 were classified as non-invasive, while grade 3 and 4 tumors were classified as invasive. Analogically, grade 1 and 2 tumors on the Hardy scale were considered non-invasive, and those with grades 3 and above were assigned to the invasive group. The patients were referred to a neurosurgeon due to their suffering from such symptoms as headache, dizziness, tinnitus, sudden visual disturbances, and sudden eyelid drooping. A total of 74 consecutive patients underwent transsphenoidal excision of the pituitary tumor via the transnasal approach. All of the operations were performed by the same neurosurgeon (R.C.) in St. Raphael’s Hospital in Krakow.

### 4.4. Statistical Analysis

Continuous variables are reported as medians with interquartile ranges (Q1–Q3) and were compared using the Mann–Whitney U test. Categorical variables are presented as counts and percentages and were compared using the Pearson Chi-square test or the Fisher exact test, as appropriate. The Fisher exact test was used when the expected frequency in any cell of the contingency table was less than 5.

To determine optimal cut-off points for PD-L1 expression (TPS) across pituitary adenoma subtypes and transcription factor expression, we applied the Liu method [70], which identifies the threshold maximizing the product of sensitivity and specificity; this was also used by Harel et al. [18]. Cut-off selection was based on binary classification (presence/absence of a tumor subtype or marker), with the TPS value yielding the highest sensitivity × specificity product being chosen as optimal.

The association between tumor invasiveness (defined as Knosp grade B or Hardy grade D) and the expression of selected biomarkers (PD-L1, P53, cyclin D1, Ki67) was assessed using simple and multivariate logistic regression. All predictors were included simultaneously in the adjusted model. Model fit was evaluated using the Hosmer–Lemeshow goodness-of-fit test, while model explanatory power was assessed using Cox–Snell and Nagelkerke pseudo-R^2^ values. Discriminative ability was measured using the area under the receiver operating characteristic curve (AUC). The results are presented as odds ratios (ORs) with 95% confidence intervals (CIs).

All analyses were conducted using available data (complete-case analysis), with no imputation applied for missing values.

Calculations were performed in Statistica v.13.3. A *p*-value of less than 0.05 was considered statistically significant.

## 5. Conclusions

Although limited by the small sample size and heterogeneity of PitNET subtypes, our study provides insights into the potential biological relevance of selected immunohistochemical markers in pituitary neuroendocrine tumors. PD-L1 expression was more frequently observed in null cell adenomas, which may suggest a distinct tumor immune microenvironment in this subtype and raise the possibility of future consideration for immunotherapeutic strategies. However, its limited diagnostic utility across other PitNET subtypes highlights the need for caution in interpretation. High cyclin D1 expression, particularly when accompanied by low P53 expression, may be associated with invasive tumor behavior. This association, while intriguing, must be interpreted conservatively due to the limited number of cases. In contrast, no clear relationship was found between Ki-67 and clinical or pathological parameters, apart from a possible association with younger patient age. Larger-scale studies with balanced representation of different PitNET subtypes are essential to confirm these findings and to better define the prognostic and therapeutic relevance of PD-L1, cyclin D1, P53, and Ki-67 expression in pituitary tumors.

## Figures and Tables

**Table 1:** Demographic, radiological, and immunohistochemical characteristics of the study group (n = 74).

Characteristic	N (%) or Mean (SD); Median (Q1–Q3)
**Age (years)**	57.4 (14.0); 60.5 (47.0–69.0)
**Gender**	Female: 29 (39.2%); Male: 45 (60.8%)
**Tumor size**	Micro: 1 (1.4%); Macro: 72 (97.3%)
Missing	1 (1.4%)
**Tumor volume (cm^3^)**	8.6 (8.9); 5.0 (3.1–10.2)
**Max tumor size (mm)**	28.6 (10.2); 25.0 (21.5–34.0)
**Transcription factors**	
PIT-1	9 (12.2%)
SF1	43 (58.1%)
TPIT	8 (10.8%)
Null cell adenoma	3 (4.1%)
Multiple PitNETs	11 (14.9%)
**Hormonal activity**	
Non-active/Active	59 (79.7%)/15 (20.3%)
**Type of PitNET**	
Gonadotroph	42 (56.8%)
Gonadotroph/lactotroph	2 (2.7%)
Corticotroph	8 (10.8%)
Lactotroph	4 (5.4%)
Somatotroph	1 (1.4%)
Thyrotroph	1 (1.4%)
Null cell adenoma	3 (4.1%)
Multiple synchronous PitNET	4 (5.4%)
Immature PIT-lineage tumor	6 (8.1%)
Mature PIT-1-lineage tumor	3 (4.1%)
**PD-L1 (TPS)**	
0%/≥1%	31 (41.9%)/35 (47.3%)
Missing	8 (10.8%)
**Proliferative factors**	
P53 < 10%/P53 ≥ 10%	34 (45.9%)/33 (44.6%)
Missing	7 (9.5%)
Ki-67 < 3%/Ki-67 ≥ 3%	65 (87.8%)/9 (12.2%)
Cyclin D1 < 10%/Cyclin D1 ≥ 10%	41 (55.4%)/29 (39.2%)
Missing	4 (5.4%)
**Knosp scale**	
Invasive/Non-invasive	38 (51.4%)/34 (45.9%)
Missing	2 (2.7%)
**Hardy scale**	
Invasive/Non-invasive	57 (77.0%)/15 (20.3%)
Missing	2 (2.7%)
**Overall invasiveness**	
Yes/No	58 (78.4%)/14 (18.9%)
Missing	2 (2.7%)

Legend: PitNET(s), Pituitary neuroendocrine tumor(s). TF, transcription factor; PD-L1, Programmed death-ligand 1; TPS, Tumor Proportion Score; PIT-1, pituitary-specific positive transcription factor 1; TPIT, T-box transcription factor. SF1, steroidogenic factor 1 transcription factor. Multiple PitNETs; tumors expressing ≥ 2 transcription factors (multiple synchronous tumor, immature PIT-1-lineage tumor, mature Pit-1-lineage tumor, gonadotroph/lactotroph).

**Table 2:** PitNET characteristics according to the expression levels of PD-L1 (n = 66, no date: 8 cases), P53 (n = 67, no date: 7 cases), Ki-67 (n = 74), and cyclin D1 (n = 70, no date: 4 cases).

	PD-L1			Ki-67			P53			Cyclin D1		
Feature	<1% (n = 31) N (%)/ Median (Q1–Q3)	≥1% (n = 35) N (%)/ Median (Q1–Q3)	*p*-Value	<3% (n = 65) N (%)/ Median (Q1–Q3)	≥3% (n = 9) N (%)/ Median (Q1–Q3)	*p*-Value	<10% (n = 34) N (%)/ Median (Q1–Q3)	≥10% (n = 33) N (%)/ Median (Q1–Q3)	*p*-Value	<10% (n = 41) N (%)/ Median (Q1–Q3)	≥10% (n = 29) N (%)/ Median (Q1–Q3)	*p*-Value
**Age** **(years)**	59.0 (43.0–70.0)	60.0 (52.0–68.0)	0.59 ᵠ	62.0 (50.0–69.0)	41.0 (36.0–57.0)	0.03 ᵠ	62.0 (53.0–70.0)	61.0 (45.0–67.0)	0.57 ᵠ	57.0 (49.0–68.0)	62.0 (47.0–68.0)	0.38 ᵠ
**Gender (F/M)**	12 (46.2%)/ 19 (47.5%)	14 (53.8%)/ 21 (52.5%)	0.91 ᵡ	28 (96.5%)/ 37 (82.2%)	1 (3.5%)/ 8 (17.8%)	0.08 ᵟ	13 (48.1%)/ 21 (52.5%)	14 (51.8%)/19 (47.5%)	0.73 ᵡ	17 (60.7%)/ 24 (57.1%)	11 (39.3%)/ 18 (42.9%)	0.77 ᵡ
**Tumor volume** **(cm^3^)**	4.6 (2.1–10.0)	6.2 (3.2–10.0)	0.55 ᵠ	4.8 (3.1–10.2)	7.3 (2.8–11.0)	0.96 ᵠ	5.2 (2.8–10.0)	7.3 (3.8–11.5)	0.41 ᵠ	4.3 (2.8–9.0)	8.1 (3.1–12.3)	0.31 ᵠ
**Max tumor size (mm)**	23.0 (22.5–34.0)	30.0 (22.0–34.0)	0.59 ᵠ	25.0 (22.0–33.0)	29.0 (20.0–36.0)	0.94 ᵠ	28.5 (22.5–34.0)	25.5 (20.8–33.0)	0.59 ᵠ	25.0 (21.0–34.0)	26.8 (22.3–33.0)	0.86 ᵠ
**PIT-1 (No/Yes)**	25 (42.4%)/ 6 (85.7%)	34 (57.6%)/ 1 (14.3%)	<0.05 ᵟ	57 (87.7%)/ 8 (88.9%)	8 (12.3%)/ 1 (11.1%)	1.00 ᵟ	30 (50.0%)/ 4 (57.1%)	30 (50.0%)/ 3 (42.9%)	1.00 ᵟ	35 (57.4%)/ 6 (66.7%)	26 (42.6%)/ 3 (33.3%)	0.73 ᵟ
**SF1 (No/Yes)**	16 (53.3%)/15 (41.7%)	14 (46.7%)/21 (58.3%)	0.34 ᵡ	26 (83.9%)/ 39 (90.7%)	5 (16.1%)/ 4 (9.3%)	0.48 ᵟ	15 (57.7%)/ 19 (46.3%)	11 (42.3%)/22 (53.7%)	0.36 ᵡ	21 (67.7%)/ 20 (50.0%)	9 (29.0%)/ 20 (50.0%)	0.09 ᵡ
**TPIT (No/Yes)**	28 (48.3%)/3 (37.5%)	30 (51.7%)/ 5 (62.5%)	0.71 ᵟ	59 (89.4%)/ 6 (75.0%)	7 (10.6%)/ 2 (25.0%)	0.25 ᵟ	30 (50.8%)/ 4 (50.0%)	29 (49.2%)/ 4 (50.0%)	1.00 ᵟ	35 (56.5%)/ 6 (75.0%)	27 (43.6%)/ 2 (25.0%)	0.54 ᵡ
**Null cell adenoma (No/Yes)**	30 (49.2%)/ 1 (20.0%)	31 (50.8%)/ 4 (80.0%)	0.24 ᵟ	62 (87.4%)/ 3 (100.0%)	9 (12.6%)/ 0 (0.0%)	1.00 ᵟ	32 (49.2%)/ 2 (100.0%)	33 (50.8%)/ 0 (0.0%)	0.49 ᵟ	39 (57.4%)/ 2 (100.0%)	29 (42.6%)/ 0 (0.0%)	0.51 ᵟ
**Multiple PitNETs (No/Yes)**	25 (44.6%)/ 6 (60.0%)	31 (55.4%)/ 4 (40.0%)	0.72 ᵟ	56 (86.2%)/ 9 (81.8%)	7 (13.9%)/ 2 (18.2%)	0.62 ᵟ	29 (50.0%)/ 5 (55.6%)	29 (50.0%)/ 4 (44.4%)	0.24 ᵟ	34 (57.6%)/ 7 (63.6%)	25 (42.4%)/ 4 (36.4%)	1.00 ᵟ
**Type of PitNET**			-			-			-			-
Gonadotroph	17 (44.7%)	21 (55.3%)		39 (92.8%)	3 (7.1%)		19 (47.5%)	21 (52.5%)		20 (51.3%)	19 (48.7%)	
Gonadotroph/Lactotroph	0 (0.0%)	2 (100.0%)		2 (100.0%)	0 (0.0%)		1 (100.0%)	0 (0.0%)		2 (100.0%)	0 (0.0%)	
Corticotroph	3 (37.5%)	5 (62.5%)		6 (75.0%)	2 (25.0%)		4 (50.0%)	4 (50.0%)		6 (75.0%)	2 (25.0%)	
Lactotroph	3 (100.0%)	0 (0.0%)		3 (75.0%)	1 (25.0%)		2 (50.0%)	2 (50.0%)		3 (75.0%)	1 (25.0%)	
Somatroph	1 (100.0%)	0 (0.0%)		1 (100.0%)	0 (0.0%)		0 (0.0%)	0 (0.0%)		0 (0.0%)	1 (100.0%)	
Thyrotroph	1 (100.0%)	0 (0.0%)		1 (100.0%)	0 (0.0%)		1 (100.0%)	0 (0.0%)		1 (100.0%)	0 (0.0%)	
Null cell adenoma	0 (0.0%)	3 (100.0%)		3 (100.0%)	0 (0.0%)		2 (100.0%)	0 (0.0%)		2 (100.0%)	0 (0.0%)	
Multiple synchronous PitNET	1 (33.3%)	2 (66.7%)		3 (75.0%)	1 (25.0%)		2 (50.0%)	2 (50.0%)		2 (50.0%)	2 (50.0%)	
Immature PIT-lineage tumor	4 (80.0%)	1 (20.0%)		5 (83.3%)	1 (16.6%)		2 (40.0%)	3 (60.0%)		4 (66.7%)	2 (33.3%)	
Mature PIT-lineage tumor	1 (50.0%)	1 (50.0%)		2 (66.6%)	1 (33.3%)		1 (50.0%)	1 (50.0%)		1 (33.3%)	2 (66.7%)	
**Hormonal** **activity** Non-active Active	23 (43.4%) 8 (61.5%)	30 (56.6%) 5 (38.5%)	0.24 ᵡ	53 (89.8%) 12 (80.0%)	6 (10.2%) 3 (20.0%)	0.38 ᵟ	25 (46.3%) 9 (69.2%)	29 (53.7%) 4 (30.8%)	0.14 ᵡ	30 (54.5%) 11 (73.2%)	25 (45.5%) 4 (26.6%)	0.19 ᵡ
**Knosp scale** Invasive Non-invasive	18 (51.4%) 16 (53.3%)	15 (45.5%) 18 (58.1%)	0.31 ᵡ	34 (89.5%) 29 (85.3%)	4 (10.5%) 5 (14.7%)	0.73 ᵟ	18 (51.4%) 16 (53.3%)	17 (48.6%) 14 (46.7%)	0.88 ᵡ	22 (59.46%) 18 (58.1%)	15 (40.5%) 13 (41.9%)	0.91 ᵡ
**Hardy scale** Invasive Non-invasive	23 (45.1%) 8 (61.5%)	28 (54.9%) 5 (38.5%)	0.29 ᵡ	51 (89.5%) 12 (80.0%)	6 (10.5%) 3 (20.0%)	0.38 ᵟ	28 (53.8%) 6 (46.2%)	24 (46.2%) 7 (53.8%)	0.62 ᵡ	31 (58.5%) 9 (60.0%)	22 (41.5%) 6 (40.0%)	0.92 ᵡ
**Invasiveness** **(Knosp or Hardy scale)** Non-invasive Invasive	6 (50.0%) 28 (52.8%)	4 (33.3%) 29 (55.8%)	0.16 ᵡ	11 (78.6%) 52 (89.7%)	3 (21.4%) 6 (10.3%)	0.36 ᵟ	6 (50.0%) 28 (52.8%)	6 (50.0%) 25 (47.2%)	0.86 ᵡ	9 (64.3%) 31 (57.45)	5 (35.7%) 23 (42.65)	0.64 ᵡ

Legend: ᵟ Fisher exact test; ᵡ Pearson Chi-square test; ᵠ Mann–Whitney U test; analyses were performed excluding missing data. We analyzed the values of the examined factors in tumors that, according to the WHO, have the potential for aggressive behavior [5]. In our study, there were two cases of Crook’s tumor (PD-L1: 0.0% and 5.0%, respectively; Ki-67: 10.0% and 1.4%, respectively, P53: 60.0% and 0.0%, respectively, cyclin D: 0.0% and 0.0%, respectively), two cases of silent corticotroph PitNETs (PD-L1: 5.5% and 0.0%, respectively; Ki-67: 0.0% and 0.5%, respectively; P53: 60.0% and 20.0%, respectively; cyclin D1: 20.0% and 0.0%, respectively), two cases of male lactotroph PitNETs (PD-L1: 0.0% and 0.0%, respectively; Ki-67: 3.2% and 1.6%, respectively; P53: 0.0% and 0.0%, respectively; cyclin D1: 0.0% and 5.0%, respectively).

**Table 3:** Associations between the expression of Ki-67, P53, and cyclin D1 and PD-L1 status (TPS 0% vs. TPS ≥ 1%).

Parameter	PD-L1 TPS 0% n (%)	PD-L1 TPS ≥ 1% n (%)	*p*-Value
**Ki-67 expression** ᵟ			0.71
<3%	28 (48.3%)	30 (51.7%)	
≥3%	3 (37.5%)	5 (62.5%)	
Missing	–	–	
**P53 expression** ᵡ			0.10
<10%	19 (57.6%)	14 (42.4%)	
≥10%	11 (36.7%)	19 (63.3%)	
Missing	1 (33.3%)	2 (66.7%)	
**Cyclin D1 expression** ᵡ			0.27
<10%	14 (41.2%)	20 (58.8%)	
≥10%	16 (55.2%)	13 (44.8%)	
Missing	1 (33.3%)	2 (66.7%)	

ᵟ Fisher Exact test; ᵡ Pearson Chi-square test; analyses were performed excluding missing data.

**Table 4:** Optimal cut-off points of PD-L1 expression according to the expression of specific transcription factors and subtypes of PitNETs while maximizing the product of the sensitivity and the specificity.

Outcome	Optimal TPS Cut-Off	Sensitivity	Specificity	AUC
**Transcription Factors**				
PIT-1	0.00	0.86	0.58	0.68
SF1	≥1.3%	0.56	0.52	0.57
TPIT	≥5%	0.63	0.59	0.50
Null Cell Adenoma	≥5%	1.00	0.59	0.75
Multiple PitNETs	≤3%	0.78	0.49	0.59
**Type of PitNET**				
Gonadotroph	≥1.3%	0.55	0.50	0.54
Corticotroph	≥5%	0.63	0.59	0.50
Lactotroph	0.00	1.00	0.56	0.78
Immature PIT-1-lineage tumor	0.00	0.80	0.56	0.67
Mature PIT-1-lineage tumor	≥85%	0.50	0.98	0.61

Legend: Quantitative variable: PDL-1 [%]. Status variable [0–1]: cell lineage (TF) and tumor type.

**Table 5:** Heatmap of PitNET types according to Ki-67 and P53 categories (percentages calculated within each PitNET type).

Type of PitNET	Ki ≥ 3 & P53 ≥ 10 (N = 6)	Ki ≥ 3 & P53 < 10 (N = 2)	Ki < 3 & P53 ≥ 10 (N = 27)	Ki < 3 & P53 < 10 (N = 32)
Gonadotroph	7.7	0	46.2	46.2
Gonadotroph/Lactotroph	0	0	0	100
Corticotroph	25	0	25	50
Lactotroph	0	25	50	25
Somatotroph	0	0	0	0
Thyrotroph	0	0	0	100
Null cell adenoma	0	0	0	100
Multiple synchronous PitNETs	25	0	25	50
Immature PIT-1-lineage tumor	0	25	75	0
Mature PIT-1-lineage tumor	0	0	50	50

The colors starting in the order: green-orange-pink-burgundy correspond to the increasing frequency (given in %) of the occurrence of a given Ki and P53 system. % is given in rows.

**Table 6:** Heatmap of PitNET types according to cyclin D1 and P53 categories (percentages calculated within each PitNET type).

Types of PitNET	Cyclin ≥ 10 & P53 ≥ 10 (N = 18)	Cyclin ≥ 10 & P53 < 10 (N = 10)	Cyclin < 10 & P53 ≥ 10 (N = 14)	Cyclin < 10 & P53 < 10 (N = 22)
Gonadotroph	26.3	23.7	26.3	23.7
Gonadotroph/Lactotroph	0	0	0	100
Corticotroph	25	0	25	50
Lactotroph	25	0	25	50
Somatroph	0	0	0	0
Thyrotroph	0	0	0	100
Null cell adenoma	0	0	0	100
Multiple synchronous PitNETs	50	0	0	50
Immature PIT-lineage tumor	40	0	20	40
Mature PIT-1-lineage tumor	50	50	0	0

The colors starting in the order: green-orange-pink-burgundy correspond to the increasing frequency (given in %) of the occurrence of a given cyclin D1 and P53 system. % is given in rows.

**Table 7:** Analysis of the effect of combinations of Ki67, P53, and Cyclin D1 on the invasiveness of PitNETs.

Features	Knosp Scale		Hardy Scale	
	Non-Invasive n (%)	Invasive n (%)	Non-Invasive n (%)	Invasive n (%)
Combined analysis of Ki-67 and P53				
Ki-67 ≥ 3% & P53 ≥ 10%	3 (50.0%)	3 (50.0%)	1 (16.7%)	5 (83.3%)
Ki-67 ≥ 3% & P53 < 10%	1 (50.0%)	1 (50.0%)	1 (50.0%)	1 (50.05)
Ki-67 < 3% & P53 ≥ 10%	11 (44.0%)	14 (56.0%)	6 (24.0%)	19 (76.0%)
Ki-67 < 3% & P53 < 10%	15 (46.9%)	17 (53.1%)	5 (15.6%)	27 (84.4%)
*p* value ᵡ	0.992		0.615	
Combined analysis of cyclin D1 and P53				
Cyclin D1 ≥ 10% & P53 ≥ 10%	8 (47.1%)	9 (52.9%)	5 (29.4%)	12 (70.6%)
Cyclin D1 ≥ 10% & P53 < 10%	4 (40.0%)	6 (60.0%)	0 (0.0%)	10 (100.0%)
Cyclin D1 < 10% & P53 ≥ 10%	5 (38.5%)	8 (61.5%)	2 (15.4%)	11 (84.6%)
Cyclin D1 < 10% & P53 < 10%	11 (50.0%)	11 (50.0%)	6 (27.3%)	16 (72.7%)
*p* value ᵡ	0.902		0.245	

ᵡ Pearson Chi-square test; analyses were performed excluding missing data.

## Data Availability

The datasets used and/or analyzed during the current study are available from the corresponding author upon reasonable request.
